# Temporal Variability of Antibiotics Fluxes in Wastewater and Contribution from Hospitals

**DOI:** 10.1371/journal.pone.0053592

**Published:** 2013-01-08

**Authors:** Sylvain Coutu, Luca Rossi, D. A. Barry, Serge Rudaz, Nathalie Vernaz

**Affiliations:** 1 ENAC/IIE/ECOL, École Polytechnique Fédérale de Lausanne (EPFL), Lausanne, Switzerland; 2 School of Pharmaceutical Sciences, University of Geneva, University of Lausanne, Geneva, Switzerland; 3 Swiss Center for Applied Human Toxicology, University of Geneva, Geneva, Switzerland; 4 Pharmacy Department, Geneva University Hospitals, Geneva, Switzerland; University of Illinois at Chicago, United States of America

## Abstract

Significant quantities of antibiotics are used in all parts of the globe to treat diseases with bacterial origins. After ingestion, antibiotics are excreted by the patient and transmitted in due course to the aquatic environment. This study examined temporal fluctuations (monthly time scale) in antibiotic sources (ambulatory sales and data from a hospital dispensary) for Lausanne, Switzerland. Source variability (i.e., antibiotic consumption, monthly data for 2006–2010) were examined in detail for nine antibiotics – azithromycin, ciprofloxacin, clarithromycin, clindamycin, metronidazole, norfloxacin, ofloxacin, sulfamethoxazole and trimethoprim, from which two main conclusions were reached. First, some substances – azithromycin, clarithromycin, ciprofloxacin – displayed high seasonality in their consumption, with the winter peak being up to three times higher than the summer minimum. This seasonality in consumption resulted in seasonality in Predicted Environmental Concentrations (*PECs*). In addition, the seasonality in *PECs* was also influenced by that in the base wastewater flow. Second, the contribution of hospitals to the total load of antibiotics reaching the Lausanne Wastewater Treatment Plant (WTP) fluctuated markedly on a monthly time scale, but with no seasonal pattern detected. That is, there was no connection between fluctuations in ambulatory and hospital consumption for the substances investigated.

## Introduction

Among the many types of pharmaceuticals, antibiotics receive particular attention concerning their risk to the natural environment. Antibiotics are widely prescribed worldwide [1]–[3], and thus are expected in receiving waters. Their presence in the aquatic environment is of concern as they are potentially harmful to organisms there. They are thought to foster bacterial resistance, for example [4]–[7]. Not surprisingly, recent studies on the occurrence of micropollutants in the environment include antibiotics [8]–[14].

Several field campaigns have reported fluctuations of pharmaceutical concentrations in receiving waters [9], [12], [15], [16], the magnitude of which varies with location and substance. Roig [17] reported an extensive overview of different field campaigns focused on antibiotics and other pharmaceuticals in surface waters, including Wastewater Treatment Plant (WTP) influent and effluent. Since the temporal variation of pharmaceutical concentrations is a supplementary pressure on aquatic system preservation [18]–[20], understanding of such variations is an important challenge in environmental assessment and management.

Antibiotics are present in both urban and rural environments. For the latter, their concentrations are often driven by veterinary use [14], [21]. In urban settings, antibiotic concentrations in wastewater result from ambulatory and hospital consumption [22], [23]. As a consequence, consumption data are needed to estimate their concentration. Several studies attempted to estimate wastewater pharmaceutical concentrations using sales data and the Predicted Environmental Concentration Model (*PEC*) [22]–[27]. These studies were not focused on short-term fluctuations as they considered only annual sales data. At present, no study has considered seasonality in consumption of antibiotics in urban settings.

Here, we first investigated seasonality of antibiotic concentrations in wastewater based on monthly sales data and the *PEC* model. Second, the fraction of antibiotics originating from hospitals in the total load found in wastewater was examined, again based on monthly consumption. The study considered nine substances – azithromycin, ciprofloxacin, clarithromycin, clindamycin, metronidazole, norfloxacin, ofloxacin, sulfamethoxazole and trimethoprim – using data for the city of Lausanne, Switzerland.

## Materials and Methods

### The WTP Basin

**Figure 1:**
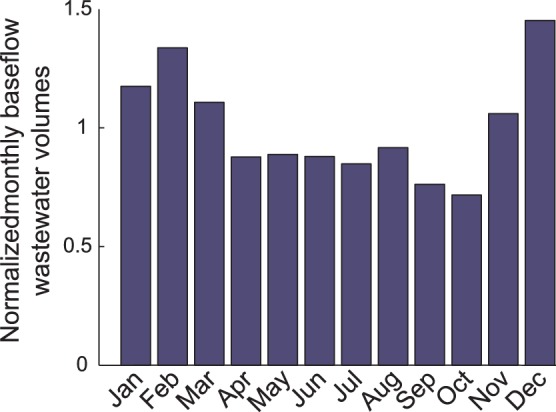
Monthly variations of dry weather flow at the Vidy Bay WTP inlet. Flow is normalized to the annual mean flow, which is 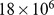 m^3^. The monthly baseflow fluctuates between +40/−25% of the mean.

The study was conducted on the main WTP catchment of Lausanne, Switzerland. This WTP serves 200′000 residents, and is located at Vidy Bay, on the shore of Lake Geneva. The annual total wastewater volume treated is approximately 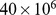 m^3^. The monthly water volume received at the WTP varies seasonally, as can be seen in Figure 1, which illustrates the dynamics of the monthly dry weather water volume at the Vidy Bay WTP inlet.

These monthly dry weather wastewater data were provided by Lausanne public authorities [28]. A seasonal trend can be observed with higher volumes in summer than in winter. Similar behaviour in WTP dry weather monthly flow volumes was observed in other studies [29], [30].

**Figure 2:**
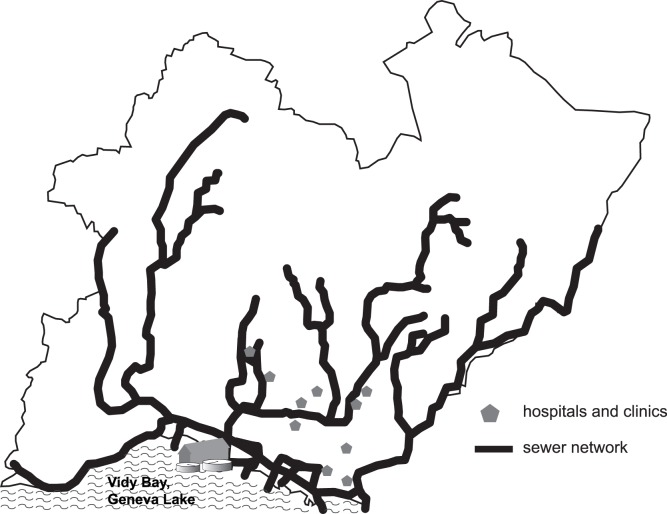
Schematic view of the Vidy Bay WTP basin considered in this study. The main pipes of the collection system are illustrated with bold lines. Hospitals and clinics for acute somatic treatment are located using grey pentagons. The total number of hospital beds in the basin is about 1600, which corresponds to a total of approximately 8 beds per 1000 inhabitant.

In our approach, we considered only hospitals or clinics performing acute somatic treatments, as such establishments dispense antibiotic prescriptions. Ten hospitals were identified, for a total of 1600 beds. Their spatial distribution on the WTP basin is illustrated in Figure 2.

### Data Source

The study is based on data from ambulatory (pharmacies) sales and hospital dispensaries. These data are considered as accurate measures of consumption. Antibiotics are prescription drugs, and are pathology-specific. Thus, the quantities prescribed should match their consumption, and sales should match the amount prescribed. For antibiotics, this connection is supported by the high patient compliance in the case of antibiotic treatment [31]. This assumption was also retained by Le Corre et al. [27] for an Australian case study. In consequence, consumption data in this text will refer to sales data. Data sources were OFAC (Professional Cooperative of Swiss Pharmacist: www.ofac.ch/, site last accessed in May 2012) and CHUV (Centre Hospitalier Universitaire Vaudois: www.chuv.ch/, site last accessed in May 2012) at a monthly resolution for a five years period (2006–2010). Data were obtained for nine antibiotics: azithromycin, ciprofloxacin, clarithromycin, clindamycin, metronidazole, norfloxacin, ofloxacin, sulfamethoxazole and trimethoprim. As trimethoprim and sulfamethoxazole are co-prescribed as a single product, they were in this study considered as a single compound named sulf/trim.

### Downscaling of Ambulatory Antibiotic Sales

This study used 2006–2010 monthly sales data obtained for the whole of the Canton of Vaud (670,000 inhabitants), as provided by OFAC. These cantonal data were downscaled to the WTP drainage area in two steps. First, data scaled to Lausanne were obtained on a per-person base, assuming that consumption was uniform over the canton. Second, since only 80% of pharmacies are affiliated with OFAC, the data were corrected by a factor of 1/0.8.

### Upscaling of Hospital Antibiotic Usage

The monthly amounts of antibiotics dispensed at Vaud’s main hospital (CHUV), located in Lausanne, were provided by the CHUV pharmacy. Corresponding data for other Lausanne hospitals could not be obtained. Total hospital consumption in the Vidy Bay WTP basin was thus obtained from the CHUV data by extrapolation to the number of beds using:
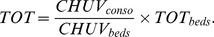
(1)


**Figure 3:**
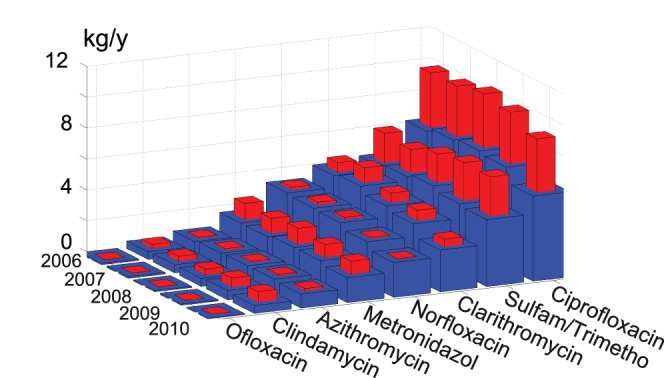
Annual antibiotics sales estimated for the city of Lausanne. Blue: ambulatory sales, red: total sales. From one year to the next, the contribution of hospitals to the total load of antibiotics consumed varies little. Likewise, ambulatory sales show little variation.

In Eq.1, the CHUV consumption, 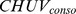, is normalized by the number of CHUV beds, 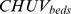 (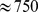), and then multiplied by the total number of hospital beds in the WTP catchment, 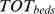 (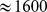). This estimation integrates indirectly the occupation rate of hospital beds, which is assumed to be identical for all hospitals. Only hospitals with acute somatic treatments were considered as they are likely to prescribe antibiotics. CHUV data covered the same period as the ambulatory antibiotics sales data. Description of antibiotics sales datasets and data processing performed are summarized in Table 1. Yearly total amounts of antibiotics used in the ambulatory and hospital domains are summarized in Figure 3.

**Table 1:** Description of the antibiotics sales datasets.

Data	Source	Period	Frequency	Population concerned	Data processing
Ambulatory	OFAC	2006–2010	monthly	Canton of Vaud	Considered number of affiliated pharmacies To downscale to the Lausanne population
Hospital	CHUV	2006–2010	monthly	CHUV pharmacy	Data were upscaled to the Lausanne hospital and bed count

Ambulatory data sales were provided by OFAC for the Canton de Vaud. These data were downscaled to the Lausanne population based on pharmacy density. Hospital data were extrapolated based on that provided by the CHUV considering the total number of hospital beds in Lausanne.

### Predicted Environmental Concentration Model (*PEC*)

With widespread awareness of the issue of pharmaceuticals in aquatic environments, public agencies like EMEA (European Medicine Agency: www.ema.europa.eu, site last accessed in March 2012) and FDA (Food and Drug Administration: www.fda.gov, site last accessed in March 2012) have supported development of predictive models of drug concentrations in receiving waters. Applications of *PEC* models occur frequently [25], [26], [32], [33]. They require pharmaceutical consumption as an input, with the output being a concentration, either in wastewater or in natural waters. However, their use with monthly antibiotic sales data has not been reported, and so here we adapted an existing model [26]:
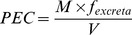
(2)where *PEC* is the model output (monthly basis), i.e., the concentration at the entrance of the WTP, *V* is the monthly volume of wastewater reaching the WTP, *M* is the monthly mass of antibiotic prescribed (sum of ambulatory and hospital prescriptions), and 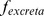 is the excretion ratio of the substance. Excretion ratios for the list of substances investigated are presented in Table 2.

**Table 2:** Excretion ratio (in % of amount consumed) in urine for the different substances investigated.

Substance	ATC	Excretionratio	Source
Azithromycin	J01FA10	12	Compendium Suisse®
Ciprofloxacin	J01MA02	55	[2]
		45–62	Compendium Suisse®
		40	[41]
Clarithromycin	J01FA09	25	[2]
Clindamycin	J01FF01	10–20	Compendium Suisse®
Metronidazole	P01AB01	40	[41]
Norfloxacin	J01MA06	30	[2]
		34	Compendium Suisse®
Ofloxacin	J01MA01/S01AX11	70	[41]
		90	Compendium Suisse®
Sulfamethoxazole	J01EE01	50	[2]
		10–30	Compendium Suisse®
		15	[42]
Trimethoprim	J01EE01	50	Compendium Suisse®

When different values were found, the highest (worst case) was selected. Data from the Compendium Suisse® can be found at http://www.kompendium.ch (site last accessed in June 2012).

In this study, we used the *PEC* model and monthly sales data to investigate fluctuations of antibiotic concentrations in the WTP influent. Monthly dry weather water volumes to the WTP were used so that predicted concentrations were not distorted by any dilution effects.

## Results and Discussion

### Periodicity in Consumption Cycle

**Figure 4:**
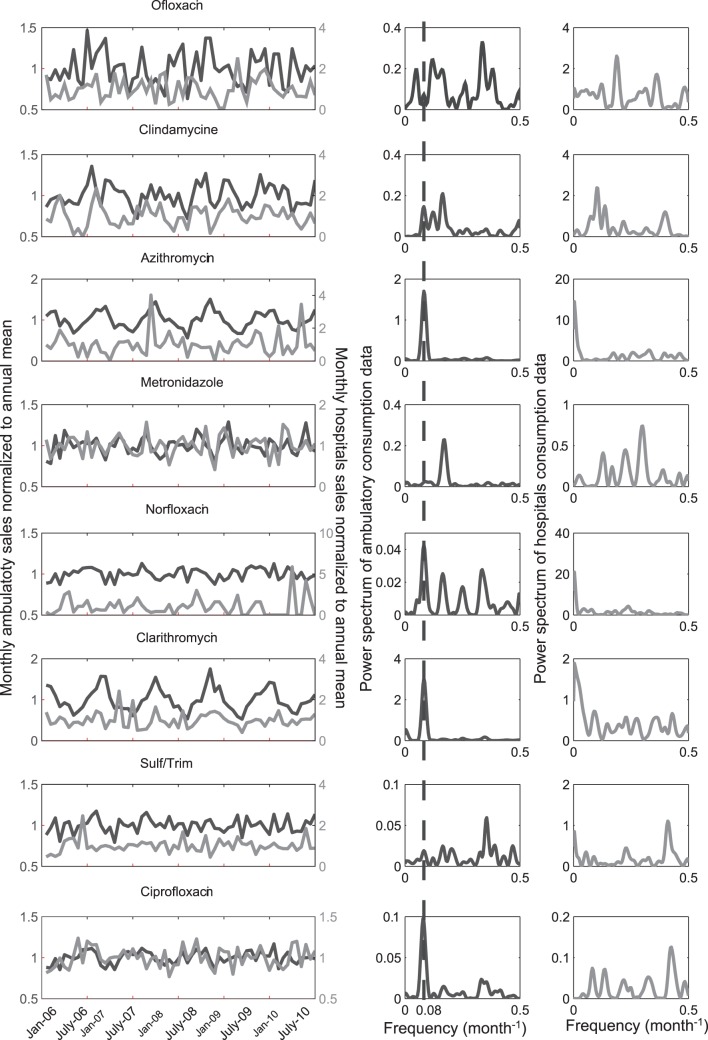
Seasonal fluctuations of antibiotic sales are shown in the left column. ambulatory (dark grey, left axis) and hospitals (light grey, right axis). The data are normalized to the annual mean. On the right side of the figure, two columns of plots show periodograms for ambulatory sales and hospital usage (left to right, respectively). The peak in the ambulatory-sales periodogram occurs at 0.08 month^−1^, i.e., a period of 12 months for these sales, indicated by the dashes.

Fluctuations in ambulatory antibiotic consumption are compared with consumption of antibiotics in hospitals for the selected substances. Figure 4 shows the monthly fluctuation of sales normalized to the annual mean for both ambulatory and hospital uses.

Three groups of substances emerge in Figure 4. The first group is comprised of azithromycin, clarithromycin and ciprofloxacin. These substances present clear seasonality of their ambulatory sales data. The visual evidence of this seasonality is supported by the periodogram of the ambulatory sales signal, with a dominant frequency found at 0.083 month^−1^, which corresponds to a 12-months period. For azithromycin and clarithromycin, the highest ambulatory sales in winter are around three times greater than the minimum consumption observed during summer. Ciprofloxacin ambulatory sales vary somewhat, between 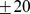
% of the annual mean. Some seasonality was also observed for norfloxacin ambulatory sales. This periodicity is absent for the hospitals uses. The second group of substances is composed of ofloxacin, clindamycin and sulf/trim. No seasonal fluctuation was found for these antibiotics, either in the ambulatory sales data or in hospital use. Finally, metronidazole was found to have a clear six months period in its ambulatory sales.

Differences in seasonal cycles for antibiotics sales data can be explained by considering their different therapeutic uses. The substances with high seasonality in ambulatory sales (azithromycin, clarithromycin and ciprofloxacin) are mainly prescribed for airway infections (i.e., bronchitis and pneumonia) and infections affecting the throat, nose and ears (pharyngitis, sinusitis, earache). The seasonality of the pathologies these substances treat explains the observed seasonality in ambulatory sales data. The other substances, for which there is no seasonal pattern in ambulatory sales, have more diverse pathologies. In many cases, they are used for non-seasonal diseases, typically infections of skin, bones, joints, urinary-genital tract, stomach and peritonitis.

### Hospital Consumption

**Figure 5:**
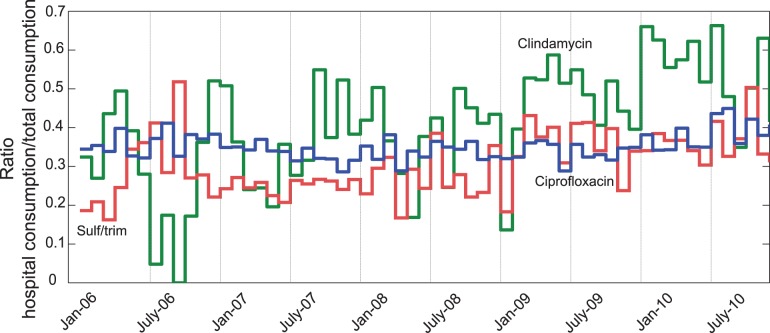
Ratio of hospital use to total use for the Lausanne WTP basin. Results are presented for clindamycin (green), trimethoprim (red) and ciprofloxacin (blue). This ratio shows considerable volatility, depending on the month and substance considered.

Previous studies that evaluated the proportion of hospital-sourced pharmaceuticals to the total WTP load typically used a fixed figure [34]–[37]. This figure was often obtained from comparison of measurements of hospital effluent and WTP influent. Recently, Le Corre et al. [27] suggested calculating this ratio from sales data, but the study is limited by the fact that authors could only provide annual data. Yet, figure 5 shows that monthly variability of this ratio can be significant.

Our data set do not reveal seasonal periodicity in the ratio of antibiotics originating from hospitals. This is explained by the seemingly random (i.e., aperiodic) behaviour of hospital antibiotic consumption, which dominates over any periodic seasonal consumption (Table 3). For some substances (e.g., ofloxacin and norfloxacin), the monthly ratio remains constantly low, with little fluctuation. On the other hand, it is difficult to draw any conclusion on the monthly variation of the hospital ratio for other substances. A specific case that can be identified is ciprofloxacin, which has a high mean annual ratio of hospital use (35%), but low monthly fluctuation (see Table 4 and Figure 5). In general, these results suggest that hospital antibiotic use is largely disconnected from non-hospital use, perhaps due to different protocols used for hospital and non-hospital patients. Another possibility is that drugs are used to treat different diseases in hospitals than in the community.

**Table 3:** Correlation coefficient of ambulatory and hospitals sales.

Substance	R
Azithromycin	0.02
Clarithromycin	0.27
Clindamycin	0.35
Ciprofloxacin	0.17
Metronidazole	0.17
Norfloxacin	0.58
Ofloxacin	0.03
Sulf/trim	0.01

R is the correlation coefficient. Consumption in hospitals and ambulatory do not correlate.

**Table 4:** Minimum/Mean/Maximum ratio (in %) of antibiotics originating from hospitals found Lausanne wastewater, calculated from 5 years of monthly consumption data.

	Ratio (%)		
Substance	Minimum	Mean	Maximum
Azithromycin	0	6.5	33.2
Clarithromycin	7.7	15.2	41.9
Clindamycin	0	40.7	66.3
Ciprofloxacin	28.6	35.1	44.9
Metronidazol	18.3	34.5	46.6
Norfloxacin	0	2.7	10.0
Ofloxacin	0	0.6	2.2
Sulf/trim	16.2	30.5	51.8

Verlicchi et al. [38] summarized existing literature estimates of ratios of hospital contribution to WTP effluent. Their summary includes the studies of [34], [36], [37], [39], [40]. Their work confirms the high spatial heterogeneity of the contribution of hospitals to the total load in WTP influent. Our study shows that temporal heterogeneity exists also. However, little variability was observed from one year to the next in the contribution of hospitals to the total antibiotic load consumed over the WTP basin (Figure 3), whereas these fluctuations can be marked when observed at a monthly time scale (Table 4). As a consequence, field campaigns that aim to estimate hospital pharmaceutical contributions to WTPs from comparison of measurements in hospital effluent and WTP influent need to account for this variability in hospital releases.

### Modelled Concentrations

**Figure 6:**
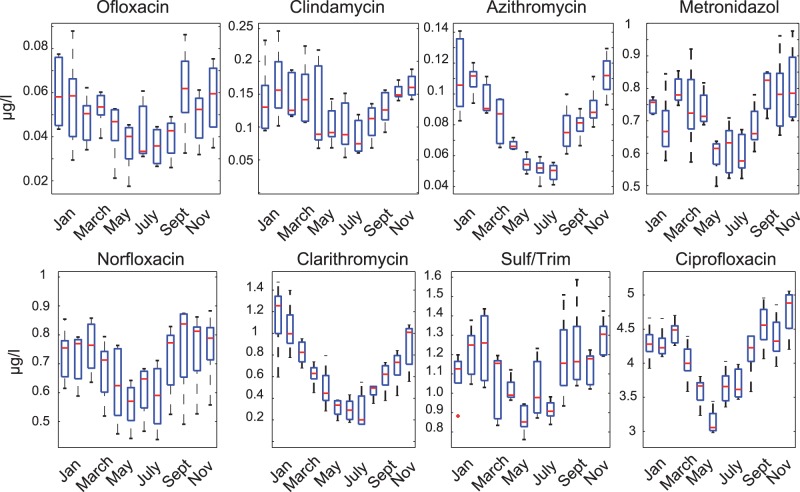
Computed monthly *PEC* at WTP inlet (one box per month). Each box was obtained from five monthly total sales data (total of 5 y of data available, giving 5 sets of each month). The red line locates the median of the five months considered. Upper and lower box limits correspond to the 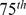 and 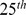 percentiles, respectively. Upper and lower whiskers correspond to the last datum being within 1.5IQR of the higher and lower quartile, respectively. IQR is the interquartile range, i.e., the difference between the upper and lower quartiles.

Here, *PEC* was calculated for WTP influent using Eq.2. Box plots of the results built from the 5-y monthly total antibiotic consumption for the Vidy Bay WTP catchment are presented in Figure 6 for the eight substances investigated. Monthly water volumes for computation of concentrations were obtained using monthly dry weather wastewater coefficients (see Figure 1).

As presented in Figure 1, monthly dry weather wastewater flow at the WTP inlet follows a periodic fluctuation (+40/−25% around annual mean). Seasonality was also observed for consumption of some antibiotics above, in particular for azithromycin, azithromycin, clarithromycin and ciprofloxacin. However, antibiotic consumption and wastewater flow tend to be out of phase. Antibiotic consumption is generally higher in winter and lower in summer, whereas flow of wastewater is lower in winter and higher in summer. This leads to an increase in the amplitude of the concentration variation predicted at the WTP inlet. The maximum seasonal effect of consumption on 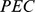 is observed for clarithromycin, with a maximum concentration predicted in January around four times greater than the minimum expected in August. This suggests that seasonality in drug consumption alone does not explain the observed fluctuations in pharmaceutical concentrations [9], [12], [15], [16]. As a consequence, this fluctuation would be equally present for all sort continuous (not rain-driven) urban sources of pollution (ammonium, cleaning products, etc.).

The 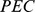 model used in this study is obviously limited by the fact that it is built upon the strong assumption of conservative mass transfer of substances from excretion to the WTP entrance. Yet, this type of modelling approach is used often in risk prioritization studies [25]–[27], [32], [33]. Typically, the 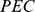 obtained from annual data is compared to a threshold effect value (for instance, the Predicted No Effect Concentration: PNEC). The substance is considered as a risk if the threshold value is exceeded. However, we highlighted in this study that several substances could show sizable fluctuations in their environmental concentrations, due to the combination of patterns that govern consumption and flow at the monthly time scale. These fluctuations will affect directly the results of the existing risk assessment methodologies. Indeed, it is now generally established that the risk of a substance does not depend solely on its average concentration but also on its concentration dynamics, which has so far not been considered [19], [20].

### Conclusion

In conclusion, this study has revealed important facts regarding antibiotic consumption as a source of environmental pollution. Some antibiotics have clear seasonal ambulatory consumption depending on their therapeutic use. Seasonality was not evident in hospital consumption. The contribution of hospitals to the total load of substances reaching the WTP is strongly dependent on time scale considered. The seasonality of ambulatory antibiotic prescriptions can be used to infer seasonality in concentrations at the WTP inlet. Yet, the variability of wastewater flow should also be considered. Seasonality in wastewater flow was found to be out-of-phase with the antibiotic fluctuation, leading to an increased amplitude of concentration fluctuations at the WTP. Prioritization studies that assess the potential risk of antibiotics or other pharmaceuticals for the environment should consider these fluctuations in their approach.

The assessment of antibiotic concentrations into wastewater from detailed sales data reduces cost and uncertainties that are usually associated to field experimental campaigns. Generally, however, detailed pharmaceutical sales data remains difficult to obtain. To investigate the time scale (month, day, hour) that drives concentration fluctuation of drugs in the environment, long-term field experimental campaigns remain mandatory.
